# How can we evaluate the cost-effectiveness of health system strengthening? A typology and illustrations

**DOI:** 10.1016/j.socscimed.2018.10.030

**Published:** 2019-01

**Authors:** K. Hauck, A. Morton, K. Chalkidou, Y-Ling Chi, A. Culyer, C. Levin, R. Meacock, M. Over, R. Thomas, A. Vassall, S. Verguet, P.C. Smith

**Affiliations:** a Department of Infectious Disease Epidemiology, School of Public Health, Imperial College London, Medical School Building, St Mary's Campus, London, W2 1PG, United Kingdom; b Department of Management Science, Strathclyde Business School, University of Strathclyde, 199 Cathedral Street, Glasgow, G4 0QU, United Kingdom; c Center for Global Development, Department of Infectious Disease Epidemiology, School of Public Health, Imperial College London, Medical School Building, St Mary's Campus, London, W2 1PG, United Kingdom; d Global Health and Development Group, Department of Infectious Disease Epidemiology, School of Public Health, Imperial College London, Medical School Building, St Mary's Campus, London, W2 1PG, United Kingdom; e Department of Economics and Related Studies, University of York, Heslington, York, YO10 5DD, United Kingdom; f Department of Global Health, University of Washington, NJB Box #359931, 325 Ninths Avenue, Seattle, WA, 98104, USA; g Centre for Primary Care, The University of Manchester, 4.311 Jean McFarlane Building, Oxford Road, Manchester, M13 9PL, United Kingdom; h Center for Global Development, 2055 L Street NW, Fifth Floor, Washington, DC, 20036, USA; i Department of Health Policy, London School of Economics and Political Science, Cowdray House, Houghton Street, London, WC2A 2AE, United Kingdom; j Department of Global Health and Development, London School of Hygiene and Tropical Medicine, Keppel Street, London, WC1E 7HT, United Kingdom; k Department of Global Health and Population, Harvard T.H. Chan School of Public Health, 665 Huntington Avenue, Boston, MA, 02115, USA; l Imperial College Business School, Imperial College London, South Kensington Campus, London, SW7 2AZ, United Kingdom

**Keywords:** Health system strengthening, Cost-effectiveness analysis, Economies of scope, Integrated service delivery, Spillover effects, Horizontal health care programs, Constraints, Healthcare delivery platforms

## Abstract

Health interventions often depend on a complex system of human and capital infrastructure that is shared with other interventions, in the form of service delivery platforms, such as healthcare facilities, hospitals, or community services. Most forms of health system strengthening seek to improve the efficiency or effectiveness of such delivery platforms. This paper presents a typology of ways in which health system strengthening can improve the economic efficiency of health services. Three types of health system strengthening are identified and modelled: (1) investment in the efficiency of an existing shared platform that generates positive benefits across a range of existing interventions; (2) relaxing a capacity constraint of an existing shared platform that inhibits the optimization of existing interventions; (3) providing an entirely new shared platform that supports a number of existing or new interventions. Theoretical models are illustrated with examples, and illustrate the importance of considering the portfolio of interventions using a platform, and not just piecemeal individual analysis of those interventions. They show how it is possible to extend principles of conventional cost-effectiveness analysis to identify an optimal balance between investing in health system strengthening and expenditure on specific interventions. The models developed in this paper provide a conceptual framework for evaluating the cost-effectiveness of investments in strengthening healthcare systems and, more broadly, shed light on the role that platforms play in promoting the cost-effectiveness of different interventions.

## Highlights

• Health interventions which rely on joint delivery platforms become interdependent.
• Such interventions must be evaluated as a portfolio to avoid allocative inefficiency.
• There are three types of health system strengthening.

## 1 Introduction

Health system strengthening (HSS) is a critical component of global public health and international development (Frenk, 2010; Kutzin and Sparkes, 2016; Naimoli et al., 2018). There is consensus that, alongside spending on specific healthcare and public health interventions, there is a need to invest in the complex health system infrastructure of human and capital resources on which delivery of these interventions is dependent. In 2015, US$ 2.7bn of all spending on development assistance for health was spent on HSS and System Wide Approaches globally. This was 7.3% of the global development assistance on health and compared to US$ 10.8bn for HIV/AIDS, US$ 6.5bn for Newborn-and-Child Health and US$ 2.3bn for Malaria, and assistance for System-wide approaches and HSS actually declined from US$ 3bn to US$ 2.7bn between 2010 and 2015 (Dieleman et al., 2016). Yet spending on HSS may be essential if health systems are to secure the full improvements in health outcomes promised by many interventions. Despite an extensive literature on HSS, there is very little evidence on exactly what proportion of funds should be spent on HSS to maximize outcomes from constrained health budgets, or how they should be spent.

It was estimated in 2014 that around $274 billion spending on health is needed per year to reach the ambitious Sustainable Development Goals 3 targets on Women's and Children's health by 2030, of which around 75% of costs are for health system strengthening, with health workforce and infrastructure (including medical equipment) as the main cost drivers (Stenberg et al., 2014). Strengthening the governance, financing and delivery of the health system to ensure rapid progress towards the health Millenium Development Goals was estimated to cost, by 2015, an additional $36–45 bn per annum, on top of the estimated $31 billion that was spent in low-income countries in 2009 (WHO, 2009). Providing the recommended care for mothers and newborns was projected to require a larger share spent on program and systems (US$ 17.7bn) than direct programmes ($ 9.1bn) (Singh et al., 2014). There are hardly any studies that comprehensively quantify the benefits and costs of HSS and compare them against spending on specific interventions (Adam et al., 2012; Barasa and English, 2011; Berman and Bitran, 2011). The analytical problem here is that the benefits of HSS for population health improvement do not materialize directly, but indirectly through the interventions that rely on the health system for delivery.

The purpose of this paper is to develop an analytical methodology that can model the impact of HSS on the cost-effectiveness of interventions and can inform the optimal balance between spending on HSS and spending directly on interventions. The intention throughout is to retain the principle of cost-effectiveness analysis (CEA), of seeking to maximize the health benefits created by interventions subject to a fixed budget constraint (Drummond et al., 2015). However, the standard focus of CEA has been on the addition of an incremental intervention. The innovation of this paper is that we explicitly consider how a range of interventions may depend on a common service delivery ‘platform’. This paper therefore differs from standard approaches towards CEA by relaxing the requirement that the costs and benefits of interventions are independent of each other, as a result of the common dependency on the platform. We demonstrate how three types of HSS can be incorporated in the cost-effectiveness model, and we provide mathematical formulations and simplified worked examples for each type. The formulations are not intended to be immediately operational or necessarily realistic. The intention is instead to use the examples and the models to bring out the essential features of the decision problems under consideration. More realistic operational models can be developed for specific decision problems using the general principles outlined in this paper.

## 2 Background

The importance of interdependencies between health interventions is reflected in the health systems literature, which recognizes that the optimal design of a health system crucially depends on the balance between different components of the health system (Chisholm and Evans, 2010; de Savigny and Adam, 2009; Over, 1988; WHO, 2000). In this section we briefly introduce the notion of service delivery platforms, and the closely associated concept of economies of scope. We then explain how the standard approach to cost-effectiveness analysis fails to take into account these important features of many health service delivery decisions.

## 3 Health service delivery platforms

Health service delivery platforms are defined by the Disease Controls Priorities project as logistically related service delivery channels that collectively make up the organisational components of the healthcare system, and mark the point of contact between service users and healthcare providers, amongst which are included hospitals, health centres and community services, see Box 1 (Watkins et al., 2017). The definition of platforms is deliberately broad, so as to include platforms that are used to deliver health promotion and prevention interventions. From a platform perspective, HSS can be defined as investments in specific inputs and infrastructures such as supply chains, clinical laboratories, physical buildings, diagnostic equipment, medical staff, and data capture systems. Concern about HSS reflects the notion that investments in such components of a delivery platform can yield benefits across a wide range of interventions that rely on the platform. It is this mutual dependency that creates the rationale for a system perspective (Smith and Yip, 2016). There are however few existing evaluative frameworks that take much consideration of such interdependency.

Box 1 **Five types of health service delivery platforms, Disease Control Priorities Project (3rd edition)**

***Population-based health interventions:*** This platform captures all non-personal or population-based health services, such as mass media and social marketing of educational messages, as typically delivered by public health agencies.

***Community services:*** The community platform encompasses efforts to bring health care services to patients, meeting people where they live. It includes a wide variety of delivery mechanisms. Specific sub-platforms include the following: health outreach and campaigns (such as vaccination campaigns, mass deworming, individual education, and counselling); schools (including school health days); community health workers, who may be based primarily in the community but also connected to first-level care providers, with ties to the rest of the system.

***Health centres:*** The health centre level captures two types of facility. The first is a higher-capacity health facility staffed by a physician or clinical officer and often a midwife to provide basic medical care, minor surgery, family planning and pregnancy services, and safe childbirth for uncomplicated deliveries. The second is a lower-capacity facility (for example, health clinics, pharmacies, dental offices, and so on) staffed primarily by a nurse or mid-level health care provider, providing services in less-resourced and often more remote settings.

***First-level hospitals:*** A first-level hospital is a facility with the capacity to perform surgery and provide inpatient care. This platform also includes outpatient specialist care and routine pathology services (such as newborn screening) that cannot be feasibly delivered at lower levels.

***Referral and specialized (second- and third-level) hospitals:*** This platform includes not only centralized, general referral hospitals with the capacity to provide secondary and tertiary care, but also specialty facilities (for example, specialized cancer-, cardiac-, and tuberculosis-related hospitals). The latter may be distinct facilities or specialized units within referral hospitals.

Alt-text: Box 1

**Source:** Watkins, D., D. Jamison, and A. Mills. "Universal health coverage and essential packages of care", in: Disease Control Priorities. New York, NY: The World Bank (2017).

## 4 Economies of scope

The idea of a platform is closely related to the notion of economies of scope, which suggest that the production of two interventions by the same entity is more cost effective than separate production, see Box 2. The presence of the shared platform leads to a reduction in costs (or improvement in benefits) compared with separate delivery of the interventions. There is weak and conflicting evidence on the presence of economies of scope in primary and secondary healthcare organizations in LMICs. Secondary care (hospital) studies focus on three angles (Barnum and Kutzin, 1993): (1) specialization on specific services or patient groups versus delivery of a broad range of services (Flessa et al., 2011; Rosenman and Friesner, 2004; Wholey et al., 1996), (2) integration of inpatient with outpatient care (Bitran-Dicowsky and Dunlop, 1989; Custer and Willke, 1991; Wouters, 1993), and (3) comparative analysis of first-level and referral hospitals, for example district versus provincial hospitals (Anderson, 1980; English et al., 2006; Weaver and Deolalikar, 2004). More recently, studies analyse economies of scope at primary care (health centre) level. The majority of studies focus on integration of HIV/AIDS prevention and treatment with other primary care services, specifically reproductive health or family planning services (Church et al., 2017; Das et al., 2007; Obure et al., 2016), tuberculosis services (Koenig et al., 2008), or paediatric care and vaccinations (Wilson, 2015). For a review see Sweeney et al. (2012).

Box 2 **Economies of Scope**

Economies of Scope (EoS) are present when $c_{1+2}\left(x_{1}+x_{2}\right)<c_{1}\left(x_{1}\right)+c_{2}\left(x_{2}\right)$ or $b_{1+2}\left(x_{1}+x_{2}\right)>b_{1}\left(x_{1}\right)+b_{2}\left(x_{2}\right)$. This means total costs of providing intervention 1 and 2 jointly are lower than providing them separately, or alternatively the benefits of providing intervention 1 and 2 jointly are higher than providing them separately. Diseconomies of scope are present if $c_{1+2}\left(x_{1}+x_{2}\right)>c_{1}\left(x_{1}\right)+c_{2}\left(x_{2}\right)\;$ or $b_{1+2}\left(x_{1}+x_{2}\right)<b_{1}\left(x_{1}\right)+b_{2}\left(x_{2}\right)$ . The magnitude of (dis-) economies of scope can be calculated by

$\;EoS_{c}=\frac{{\text{c}}_{1}\left({\text{x}}_{1}\right)+{\text{c}}_{2}\left({\text{x}}_{2}\right)-{\text{c}}_{1+2}\left({\text{x}}_{1}+{\text{x}}_{2}\right)}{{\text{c}}_{1+2}\left({\text{x}}_{1}+{\text{x}}_{2}\right)}$ or $\;EoS_{b}=\frac{{\text{b}}_{1+2}\left({\text{x}}_{1}+{\text{x}}_{2}\right)-{\text{b}}_{1}\left({\text{x}}_{1}\right)-{\text{b}}_{2}\left({\text{x}}_{2}\right)}{{\text{b}}_{1+2}\left({\text{x}}_{1}+{\text{x}}_{2}\right)}$

So for example, if the costs of providing intervention 1 are $c_{1}\left(x_{1}\right)=\$1.5m$, the costs of providing intervention 2 are $c_{2}\left(x_{2}\right)=\$2.4m$, and the costs of providing the two interventions together are $c_{1+2}\left(x_{1}+x_{2}\right)=\$3m$, then the value of the economies of scope can be calculated as

$$\;EoS_{c}=\frac{1.5+2.4-3}{3}=\frac{0.9}{3}=0.3$$

If the benefits of providing intervention 1 are $b_{1}\left(x_{1}\right)=600\;Quality-adjusted\;life\;years\;\left(QALYs\right)$, the benefits of providing intervention 2 are $c_{2}\left(x_{2}\right)=1500\;QALYs$, and the benefits of providing the two interventions together are $b_{1+2}\left(x_{1}+x_{2}\right)=3000\;QALYs$, then the value of the economies of scope can be calculated as

$$\;EoS_{b}=\frac{3000-600-1500}{3000}=\frac{900}{3000}=0.3$$

This measure indicates the savings or gains of joint compared to separate production, as a percentage of joint production. It shows that providing both interventions together results in cost savings or outcome increases of 30%.

The concept of economies of scope was originally developed to explain the benefits of diversification of multi-product firms. A hospital is the prime example of a multiproduct firm with its substantial economies of scope across the diverse healthcare interventions it delivers. Hospitals provide an infrastructure of general services and inputs (such as administration, laboratories, diagnostic equipment, theatres, etc) which are used and shared across a wide range of interventions. The costs of the platform can consequently be shared across all these interventions, resulting in efficiency gains that can be used to reduce aggregate costs or increase aggregate outcomes.

Alt-text: Box 2

Surprisingly, most studies find either no or very minor economics of scope on hospital or health centre level, with some even suggesting that there are dis-economies of scope, implying that joint implementation is less cost-effective than separate implementation. The difficulty of finding empirical evidence of economics of scope is probably explained by the problem that they materialize only if the shared platform has the capacity to deliver the two interventions; if that is not the case, interdependencies may result in incidental reductions in outcomes or increases in costs of other interventions delivered by the same platform although it is difficult to find empirical evidence of these impacts (Gallagher et al., 2018; Kristensen et al., 2015). They can be avoided if the introduction of a new intervention comes alongside investments in the delivery platform. Indeed, studies on economies of scope between interventions delivered by a newly established platform of integrated community services do find evidence of economies of scope. For example, the costs of integrating home-based testing and counselling for HIV with those for non-HIV conditions (hypertension, diabetes, malaria) were found to be very small, indicating potential economies of scope though the studies did not explicitly investigate this (Chang et al., 2016; Janssens et al., 2007). A 7-day integrated mobile testing campaign that targeted HIV, malaria and diarrhoea was found to be cost-saving (Kahn et al., 2012).

## 5 Standard cost-effectiveness analysis

Standard CEA assumes that interventions are independent, in the sense that the costs (and benefits) of each intervention remain unchanged whatever other interventions are implemented. This is because a common focus of CEA in high-income countries is on situations where a single new technology, most commonly a novel drug, is being introduced into a health system, but the delivery platforms remain essentially unchanged (Drummond et al., 2015; Vassall et al., 2017). The standard approach can be represented as a simple mathematical programme, the solution of which requires that interventions (assumed to be independent) are selected for adoption in ascending order of cost-effectiveness until the budget is exhausted (Crown et al., 2017). This formulation ignores many practical complications but serves as a useful starting point. The basic model is a ‘knapsack’ problem for choosing the welfare-maximizing set of interventions with a limited budget *B*.

$$maximize\sum_{i}x_{i}v_{i}\;subject\;to\sum_{i}x_{i}c_{i}\leq B\;;\;$$

$$all\;0\leq x_{i}\leq 1$$

Each candidate intervention *i* for adoption yields incremental benefits *v*_*i*_ and incurs incremental costs *c*_*i*_. Benefits *v*_*i*_ can be measured in natural units (e.g. infections averted) or with a generic measure of health such as quality- or disability-adjusted life-years. The decision variables *x*_*i*_ indicate the proportion of intervention *i* implemented (coverage). The model is solved to find the values for *x*_*i*_ that maximize aggregate health from all interventions *v*_*i*_, subject to the budget constraint *B*. This yields the usual CEA marginality condition that interventions are accepted if $v_{i}/c_{i}\geq \lambda$, where 1/ λ is the cost-effectiveness threshold. It reflects the opportunity costs in terms of forgone health benefits, a measure of the ‘cost per unit of health benefit (e.g. cost per Quality-adjusted life year gained or cost per Disability-adjusted life year averted) forgone’. The smaller $v_{i}/c_{i}$, the poorer is the cost-effectiveness of intervention *i*. In practice, the costs *c*_*i*_ may include expenditure on health system strengthening (HSS) specific to intervention *i,* or some estimate of the contribution to the costs of an existing or newly established delivery platform shared with other interventions.

Yet if several interventions rely on a delivery platform, they are implicitly interdependent, and it is often impossible satisfactorily to attribute to specific interventions the HSS costs that improve the quality or capacity of that platform. From a systems perspective, therefore, the costs and benefits of a specific intervention are often conditional on the other interventions chosen to share the common delivery platform. The existence of platforms introduces additional considerations into the standard CEA framework. A decision to implement a specific intervention may have positive or negative effects on the costs and benefits of other interventions depending on whether it helps share the costs of an underutilized existing platform, puts pressure on the capacity of an existing platform, or introduces a new platform. We will illustrate these three scenarios with worked examples, supported by mathematical formulations. Note that the interventions we have chosen for the scenarios, and their costs, are not real-world case studies and are meant for illustrative purposes only.

## 6 Typology of health system strengthening

### 6.1 HSS 1: investing in platform efficiency

Investments in an existing shared platform can improve the technical efficiency of the platform, i.e. the effectiveness with which a given set of inputs is used to produce an output. Such investments could take the form, for example, of new information systems, workforce training, or improved laboratory facilities. The efficiency improvement will in turn affect the cost-effectiveness of existing interventions that use the platform, by either improving service quality and thus health outcomes, or reducing unit costs, or both. Improvements in the quality of the platform may improve the cost-effectiveness of many interventions, a classic example of economies of scope. They may also change the relative ranking of interventions with respect to their cost-effectiveness.

A previous study has shown how it is possible to identify the optimal balance between investments in interventions and efficiency-improving HSS using mathematical programming methods (Morton et al., 2016). The innovation is to introduce an additional decision variable *y* into the standard CEA formulation that indicates the expenditure on HSS. The effect of spending $*y* on HSS is to scale the effectiveness of all interventions using a platform by a dilution factor of $y^{\gamma }$, where 0<γ<1 is a parameter reflecting diminishing returns to further investments in HSS in terms of additional health gained. For example, *y* may capture the additional costs of a ‘pay for performance’ (P4P) initiative for nursing staff, imposing a cost in the form of nursing reimbursement, but also yielding more effectively delivered interventions across the board. Through the parameter y, HSS type1 is implicitly addressing the ease with which technical efficiency of a platform can be improved. The simplest case is to assume that *y* uniformly improves the effectiveness of all interventions (Morton et al., 2016). The optimization problem is then

$$maximize\;y^{\gamma }\sum x_{i}v_{i}\;subject\;to\;y+\sum x_{i}c_{i}\leq B\;;$$

$$all\;0\leq x_{i}\leq 1;\;y_{L}\leq y\leq y_{U}$$

The first term replicates the usual CEA objective with the additional HSS scaling factor $y^{\gamma }$. In this augmented model it is necessary to choose the optimal level of HSS *y* in addition to the optimal proportion of interventions *x*_*i*_, subject to the budget constraint *B*. Note that the costs of *y* must also be considered in the budget constraint, highlighting the trade-off between intervention coverage and HSS. Assuming boundary conditions do not bind, this yields the marginality condition that for accepted interventions $\frac{v_{i}}{c_{i}}\geq \frac{\gamma \sum_{i}v_{i}x_{i}}{y}$. That is, at the margin, the accepted interventions are at least as cost-effective as further investment in HSS. The HSS is an efficient way of improving the benefits (and thus the cost-effectiveness) of all interventions using the platform, in a manner that would usually have been infeasible if the interventions had not been interdependent, and effectively improves the technical efficiency with which the platform functions. This model captures interdependencies on the benefit side, but it can be readily adapted to allow for the possibility that HSS is cost reducing rather than quality improving. It can also allow for non-uniform impact of *y* on interventions, or for HSS as an incidental consequence of introducing a specific intervention that has positive impacts on several other interventions.

Consider the example in Table 1. Delivery of voluntary medical male circumcision (VMMC) to 6000 patients and basic trauma services to 9500 patients in remote facilities accrue total fixed costs of $7.5 m and average variable costs of $200 and $500 per patient respectively. Incremental health benefits per case are 7 and 5 Quality-adjusted life years (QALYs) on average. Total cost per QALY of the interventions are respectively $98 = ($484+$200)/7 and $197 = ($484+$500)/5, assuming an equal distribution of the fixed costs across patients. Both interventions are therefore within the country's current threshold of $200 per QALY.

**Table 1:** Investing in existing platforms (HSS 1).

**Existing healthcare provision**						
	**VMMC**	**Trauma**		**Total/** **Average**		
Allocation fixed costs (per case)	484	484		484	Fixed costs (total)	7,500,000
Variable costs (per case)	200	500		384	Variable costs (total)	5,950,000
Incremental benefits (QALYs per case)	7	5			Total costs	13,450,000
Number of cases	6,000	9,500		15,500	Total QALYs	89,500

**Additional $2m spent on intervention HPV**						
	**VMMC**	**Trauma**	**HPV**	**Total/** **Average**		
Allocation fixed costs (per case)	294	294	294	294	Fixed costs (total)	7,500,000
Variable costs (per case)	200	500	200	0	Variable costs (total)	7,950,000
Incremental benefits (QALYs per case)	7	5	2		Total costs	15,450,000
Number of cases	6,000	9,500	10,000	25,500	Total QALYs	10,9500

**Additional $2m spent on HSS**						
	**VMMC**	**Trauma**		**Total/** **Average**		
Allocation fixed costs (per case)	613	613		613	Fixed costs (total)	9,500,000
Variable costs (per case)	200	500		384	Variable costs (total)	5,950,000
Incremental benefits (QALYs per case)	9	7			Total costs	15,450,000
Number of cases	6,000	9,500		15,500	Total QALYs	120,500

**Total Cost/QALY**	**90**	**159**		**128**		

Threshold for adoption is a Cost/QALY ratio below $200.

The decision maker needs to make a decision between investing an additional $2 million on HPV vaccinations or HSS. An additional $2 m spent on HPV vaccinations would translate into 10,000 patients vaccinated, and generate 2 QALYs per case, with variable costs of $200 per patient. In addition, we assume that it would allow the fixed costs of the health facility platform to be shared amongst more patients, thereby reducing the cost-per-QALY of the VMMC and trauma interventions. At $247 per QALY, the HPV intervention alone would not be cost-effective. However, joint provision of the three interventions results in a lower cost-per-QALY ($141). As a result, considering the joint provision of the interventions, all three interventions would be cost-effective using the country's current threshold of $200 per QALY.

However, the additional $2 m could also be spent on HSS (see Table 1). For example, HSS may consist of introduction of an electronic patient record system, which reduces adverse events and medication side-effects, with a positive impact on delivery of care for VMMC and trauma services, assuming a uniform increase in effectiveness for this example. Note that the fixed costs increase in aggregate by the £2 m spent on HSS. The quality improvements would result in an increase of QALYs gained from 7 to 9 QALYs in the case of VMMC and from 5 to 7 QALYs for trauma services. Under this scenario, the cost per QALY for the two interventions are respectively $90 and $159; and the combined cost-per-QALY is $128.

While both HPV and HSS investment are cost effective using the country threshold ($200 per QALY), the additional $2 m would be better spent on HSS than the HPV vaccinations.

### 6.2 HSS 2: investing in platform capacity

Changes in the way an existing shared platform is used can improve the mix of services it supports, and therefore the allocative efficiency of the platform, without necessarily increasing expenditure. Allocative efficiency implies that the health system delivers the optimal range and coverage of healthcare interventions. The second type of HSS therefore arises from the joint reliance of interventions on a shared platform in which some resource constraint limits the capacity to produce the optimal quantity and range of outputs. The objective of HSS in these circumstances is to relax such capacity constraints.

There is a well-established literature on physical health system constraints that may affect the optimal range of interventions provided (Luboga et al., 2016; Wilson, 2015). Amongst the most frequently cited are the limits in the numbers and skill levels of human resources. When a new intervention is introduced that requires delivery by skilled health professionals (say doctors), this may have a detrimental impact on the cost-effectiveness of existing interventions that also depend on that scarce resource for delivery. For example, in order to accommodate the new intervention, the mix of inputs used in existing interventions may be altered by shifting tasks for those services to less skilled workers, resulting in a loss in effectiveness (Fulton et al., 2011). This unintended spillover is an unintended consequence arising from the introduction of the new intervention. Such constraints may arise not only through human resources, but also for example through fixed capacity of facilities, equipment or local financing.

HSS might therefore seek to relax the resource constraint by, for example, training more staff to fulfil skilled tasks. Following van Baal et al. (2018), a simple approach for modelling the decision problem is to impose two separate constraints, say B_L_ and B_O_ for labour and for other spending.

$$maximize\;\sum x_{i}v_{i}\;subject\;to\sum x_{i}c_{i}^{L}\leq B_{L}\;;\;\sum x_{i}c_{i}^{O}\leq B_{O};$$

$$all\;0\leq x_{i}\leq 1,$$

where $c_{i}^{L}$ and $c_{i}^{O}$ are the resource use of intervention *i.* There are now two shadow prices $\lambda_{L}\;and\;\lambda_{O}$, and the decision rule is that interventions should be accepted if $v_{i}\geq c_{i}^{L}\lambda_{L}+c_{i}^{O}\lambda_{O}$. Introducing the additional constraint B_L_ favours interventions that make relatively smaller demands on labour. The effect of relaxing constraint *B*_*L*_ via HSS is to allow more interventions to be delivered and shift towards more cost-effective interventions that could not previously be implemented because of the opportunity cost they imposed by pre-empting use of the scarce labour resource. The key investment trade-off is between relaxing the labour constraint and relaxing the other constraint. Only when the two shadow prices $\lambda_{L}\;\text{and}\;\lambda_{O}$ become equal does it become optimal to invest in general budget support, rather than in addressing the scarce constraint.

An alternative formulation would be to consider a different method of delivering treatments which requires less intensive use of the scarce resource. This requires a simple adaptation of the above mathematical programme to include a revised set of delivery methods with parameters $\left\{v_{i}^{*};c_{i}^{L*};c_{i}^{O*}\right\}$, where $c_{i}^{L*}<c_{i}^{L}$. It may often be the case that the reduced use of the scarce resource leads to lower expected benefits $v_{i}^{*}<v_{i}$. Note also that a treatment can be delivered only once, so that for all *i* the associated decision variables must satisfy $x_{i}+x_{i}^{*}\leq 1$.

Consider the example in Table 2. Second-line treatment for multi-drug resistant tuberculosis (TB) is currently delivered to 8500 patients at variable costs of $200 and fixed costs of $882 per case and $7.5 m in total. At $180, the cost-per-QALY of TB second-line treatment is below the threshold of $200 and it was therefore adopted. The country now considers the adoption of medication to treat common cardiovascular diseases (CVDs), relying on the same platform, a cadre of medically trained nurses that is currently delivering the TB therapy. There is capacity to deliver CVD treatment to a maximum of 5000 patients, at fixed costs of $1500 and variable costs of $100. However, CVD treatment would not be cost-effective at cost-per-QALY of $267 if it were implemented on its own with sole use of the platform.

**Table 2:** Investing in constrained platforms (HSS 2).

**TB 2**^**nd**^**line on its own (cost-effective, existing healthcare provision)**					
	**TB 2**^**nd**^**line**		**Total/** **Average**		
Allocation fixed costs (per case)	882		882	Fixed costs (total)	7,500,000
Variable costs (per case)	200		200	Variable costs (total)	1,700,000
Incremental benefits (QALYs per case)	6			Total costs	9,200,000
Number of cases	8,500		8,500	Total QALYs	51,000
**Total Cost/QALY**	**180**		**180**		

**CVD treatment on its own (not cost-effective)**					
		**CVD**	**Total/** **Average**		
Allocation fixed costs (per case)		1500	1500	Fixed costs (total)	7,500,000
Variable costs (per case)		100	100	Variable costs (total)	500,000
Incremental benefits (QALYs per case)		6		Total costs	8,000,000
Number of cases		5,000	5,000	Total QALYs	30,000
**Total Cost/QALY**		**267**	**267**		

**Joint provision of TB 2**^nd^**line and CVD treatment without HSS 2 (capacity constraint)**					
	**TB 2**^nd^**line**	**CVD**	**Total/** **Average**		
Allocation fixed costs (per case)	714	714	714	Fixed costs (total)	7,500,000
Variable costs (per case)	200	100	152	Variable costs (total)	1,600,000
Incremental benefits (QALYs per case)	4	6		Total costs	9,100,000
Number of cases	5,500	5,000	10,500	Total QALYs	52,000
**Total Cost/QALY**	**229**	**136**	**175**		

**Joint provision of TB 2**^nd^**line and CVD treatment with HSS 2 (capacity constraint relaxed)**					
	**TB 2**^nd^**line**	**CVD**	**Total/** **Average**		
Allocation fixed costs (per case)	704	704	704	Fixed costs (total)	9,500,000
Variable costs (per case)	200	100	27	Variable costs (total)	2,200,000
Incremental benefits (QALYs per case)	6	6		Total costs	11,700,000
Number of cases	8,500	5,000	13,500	Total QALYs	81,000
**Total Cost/QALY**	**151**	**134**	**144**		

Threshold for adoption is a Cost/QALY ratio below $200.

The decision maker considers joint implementation of TB second-line and CVD treatment. At first glance, sharing the fixed costs of the platform might have the advantage that it secures a reduction of the fixed costs per case for both interventions to $714 each and a favourable cost-per-QALY ratio for CVD treatment, compared to sole implementation (see Table 2). However, the nursing constraint has negative consequences for TB second-line treatment: First, it reduces the number of patients that can be treated, and second, it reduces the quality of treatment from 6 to 4 incremental QALYs gained per patient, for example, because the nurses reduce consultation times for patients, spend less time on writing patient reports or fail to coordinate care of certain patients with other care providers, leading to increases in adverse events or side effects that are not treated in a timely manner. As a consequence, the cost-effectiveness of TB second-line treatment worsens from $180 to $229 and is now above the threshold of $200 and therefore no longer cost-effective if it were considered in isolation.

However, CVD treatment makes cost-effective use of the platform, because it can share the fixed costs of the platform with TB treatment. The 5000 patients requiring CVD treatment can now be covered at a favourable cost per QALY of $136. If considered in isolation, the less intensive TB treatment is not cost-effective with the addition of CVD treatment, but the cost-effectiveness of the two treatments must be considered jointly. The addition of CVD treatment leads to a net reduction in aggregate costs-per-QALY from $180 to $175, and so the new scenario where TB and CVD treatment are delivered in conjunction is preferable to the scenario where only TB treatment is delivered. In effect, CVD treatment allows better use to be made of the fixed constraint, notwithstanding the adverse effects for patients receiving TB treatment.

If policy makers do not want to accept the negative impact for TB second-line treatment, they can invest in the capacity of the platform (HSS 2) to reduce or relax the constraint. We assume that $2 m are spent, for example by hiring more nurses or giving them training. This allows treating the original 8500 cases with TB second-line treatment and improves the cost-effectiveness of both TB and CVD treatment. The joint cost-effectiveness increases to $144 cost-per-QALY, compared to the situation without HSS. The effect of HSS 2 is to improve the allocative efficiency of the delivery platform by reducing the impact of a binding constraint.

### 6.3 HSS 3: investing in new platforms

Finally, interdependencies can occur when an intervention introduces the need for a delivery platform that has not hitherto been in place, and that could be shared by other interventions. Investing in a new shared platform could support a number of existing or new interventions. This type of HSS differs from the other two types because it may expand the scope of interventions that can be considered for implementation. It may also alter the costs or benefits of existing interventions, leading to a reordering of interventions based on cost-effectiveness. An example for this type of HSS might be community services in the form of a new mobile HIV testing and counselling campaign, which creates a potential platform for other interventions including testing for malaria, hypertension and diabetes (Chang et al., 2016; Janssens et al., 2007; Kahn et al., 2012).

To formulate mathematically, we need to first consider a scenario without the proposed platform;

$$maximize\sum_{i\in S}x_{i}v_{i}^{S}\;subject\;to\;\sum_{i\in S}x_{i}c_{i}^{S}\leq B\;;\;$$

$$all\;0\leq x_{i}\leq 1,$$

where S is the set of all possible interventions that can be delivered without the new platform, and $v_{i}^{S}$ and $c_{i}^{S}$ are the associated benefits and costs as in standard CEA. With the new platform this becomes

$$maximize\sum_{i\in S}x_{i}v_{i}^{S}+\;\sum_{i\in P}z_{i}v_{i}^{P}$$

$$subject\;to\;C_{P}+\sum_{i\in S}x_{i}c_{i}^{S}+\sum_{i\in P}z_{i}c_{i}^{P}\;\leq B\;;$$

$$all\;0\leq x_{i}+z_{i}\leq 1;\;0\leq x_{i}\leq 1;\;0\leq z_{i}\leq 1,$$

where P is the set of interventions that could use the new platform, *z*_*i*_ are the associated decision variables, and C_P_ is the fixed cost of the platform. The health benefits obtained from the first scenario must be compared with those from the second in order to decide whether or not to invest in the platform. The additional constraints ensure that mutually exclusive interventions (reflecting interventions that could be delivered with or without the infrastructure) are selected only once. That is, each intervention *i* can only be delivered with or without the platform, or not at all. Interventions that cannot be delivered with S (or P) will be attributed zero benefits $v_{i}^{S}$ (or $v_{i}^{P})$.

A succinct way of expressing the comparison of the two models is as follows:

$$maximize\sum_{i\in S}x_{i}v_{i}^{S}+\;\sum_{i\in P}z_{i}v_{i}^{P}$$

$$subject\;to\;yC_{P}+\sum_{i\in S}x_{i}c_{i}^{S}+\sum_{i\in P}z_{i}c_{i}^{P}\;\leq B\;;$$

$$\sum_{i\in P}z_{i}\leq My$$

$$all\;0\leq x_{i}+z_{i}\leq 1;\;0\leq x_{i}\leq 1;\;0\leq z_{i}\leq 1;\;y\in \left\{0,1\right\},$$

The new decision variable *y* is set to 1 if the platform is implemented, zero otherwise. The new constraint ensures that the set of interventions in *P* can be implemented only if the platform is put in place, the constant *M* merely being a sufficiently large constant.

Table 3 gives an example. Viral load (VL) testing of patients with HIV is being considered for widespread adoption in primary care facilities. Because the test kits are not thermostable, adoption of VL testing requires investment in a new cold supply chain, with high annual fixed cost of $11 m (including annualized depreciation). The introduction of the new platform opens up the possibility of delivering Oxytocin for the prevention of postpartum haemorrhage to 12,500 women giving birth, who are currently not receiving this prevention intervention. Considered in isolation, Oxytocin is not cost-effective either at $276, and has not hitherto been considered for adoption.

**Table 3:** Investing in new platforms (HSS 3).

**Provision of Viral load testing on its own (not cost-effective)**					
	**VL test**		**Total/** **Average**		
Allocation fixed costs (per case)	1294		1294	Fixed costs (total)	11,000,000
Variable costs (per case)	200		200	Variable costs (total)	1,700,000
Incremental benefits (QALYs per case)	7			Total costs	12,700,000
Number of cases	8,500		8,500	Total QALYs	59,500
**Total Cost/QALY**	**213**		**213**		

**Provision of Oxytocin on its own (not cost-effective)**					
		**Oxytocin**	**Total/** **Average**		
Allocation fixed costs (per case)		880	880	Fixed costs (total)	11,000,000
Variable costs (per case)		500	500	Variable costs (total)	6,250,000
Incremental benefits (QALYs per case)		5	5	Total costs	17,250,000
Number of cases		12,500	12,500	Total QALYs	62,500
**Total Cost/QALY**		**276**	**276**		

**Platform shared between Viral load testing and Oxytocin**					
	**VL test**	**Oxytocin**	**Total/** **Average**		
Allocation fixed costs (per case)	524	524	524	Fixed costs (total)	11,000,000
Variable costs (per case)	200	500	379	Variable costs (total)	7,950,000
Incremental benefits (QALYs per case)	7	5		Total costs	18,950,000
Number of cases	8,500	12,500	21,000	Total QALYs	122,000
**Total Cost/QALY**	**103**	**205**	**155**		

Threshold for adoption is a Cost/QALY ratio below $200.

If the two interventions are both implemented, they share the fixed costs of the putative distribution system. That is, the $11 m cost of the platform can be shared amongst the total of 21,000 patients receiving the two interventions. Assuming an equal allocation across patients, this results in a reduction in fixed costs from $1294 (VL testing) and $880 (Oxytocin) to $524 per case, and a reduction in cost-per-QALY from $213 to $103 (VL testing) and $276 to $205 (Oxytocin). Note that this favourable cost-effectiveness can be secured only if Oxytocin is implemented alongside VL testing, sharing the fixed costs. The ‘package’ of VL testing and Oxytocin has a combined total cost of $18.95 m, which is higher than the $12.7 m required for VL testing alone. However, the package yields an aggregate cost-per-QALY of $155, below the threshold of $200, and therefore represents a good use of these additional funds, provided they can be secured.

## 7 Conclusions

Much of the HSS literature is implicitly concerned with the need to address opportunities and constraints presented by service delivery platforms. Many constraints on decision makers arise from the existence of platforms that impose an element of fixed costs on the health system (Hauck et al., 2016). Platforms provide the opportunity to exploit economies of scope, i.e. increases in cost-effectiveness of interventions arising from their joint delivery. Economies of scope materialize if a platform has some slack capacity, therefore reducing the unit opportunity costs for interventions that can use the platform. Economies of scope however cannot be exploited if a platform has constrained capacity, therefore increasing the opportunity costs of relevant interventions.

This paper has examined three classes of HSS associated with delivery platforms. They are (1) HSS that improves the technical efficiency of an existing platform and is explicitly designed to increase the cost-effectiveness of a range of existing interventions, through improved quality, reduced unit costs, or both; (2) HSS that invests in the capacity of a platform when interventions compete for a delivery platform that has limited capacity, for example due to shortages in human resources. Such shortages lead to inefficiencies because economies of scope cannot be exploited. The marginal benefits of HSS are reflected in the reduced implicit prices of the constraints. Such HSS offers the ability to shift towards more cost-effective interventions, increase access to services, and therefore improve allocative efficiency; (3) HSS that introduces a new platform to support a new intervention that may create opportunities for cost-effective delivery of other interventions that could share the platform. Economies of scope arising from joint implementation make new interventions cost-effective compared to a situation where they would be implemented on their own. The paper illustrates these three classes of HSS with simplified examples, and how they can be modelled mathematically. The principal insight is that when there is reliance on a joint platform, interventions become interdependent, and must therefore be evaluated as a portfolio (Salo et al., 2011). Myopic focus on individual interventions can result in misleading analyses when services depend on broader delivery platforms, resulting in allocative inefficiencies within the system. Each of the models presented examines the trade-off between expenditure on HSS and expenditure on direct service delivery and seeks to find an optimal balance.

With this paper, we expand on the work by Morton et al. (2016) to define the different ways in which HSS may influence implementation. As with Morton et al., we see the health system as a platform that influences multiple interventions; and can do so in several ways either by improving the efficiency of the existing shared platform (HSS 1, in line with Morton at al), by relaxing a capacity constraint (HSS 2), expanding on van Baal et al. (2018), and by providing improved coverage by expanding the platform to new populations (HSS 3).

The models developed in this paper are consistent with standard CEA but seek to model platforms and the associated cost structures more realistically. The models for HSS types 1 and 2 are more applicable in local decision-making situations, where the details of capacity constraints, service demands and other relevant factors related to interdependencies are likely to be very important. HSS type 3 will often be relevant to less incremental and more strategic decision-making. Such reforms will often require considerable investment, occur less frequently, and be taken at a more central budgetary level. However, the distinction between the models presented is to some extent artificial, because many realistic decision problems contain elements of all three types of HSS.

We assume an average level of efficiency in the use of existing resources, following usual practice in most CEA (Calxton et al., 2015). Eckermann and Pekarsky (2014) and Paulen et al. (2017) argue for the importance of considering the efficiency with which existing resources are used when assessing the cost-effectiveness of new interventions. Our paper is examining the impact of health system constraints on the conventional practice of CEA, so consideration of current use of resources is beyond the scope of the paper. Economic analysis of HSS may often be necessary when developing the range of services to be included in a health benefits package as countries make the transition towards universal health coverage. The implications of this research are that the optimal package of care may depend on the health system infrastructure in place, particularly in countries with regional differences in delivery mechanisms and system designs. Furthermore, if a uniform national health benefit package is put in place, some localities with atypical platforms may need higher levels of reimbursement so that they are able to deliver the package or may need assistance in reconfiguring their systems so as to maximize the efficiency with which they can deliver the chosen package in the future.

**Fig. 1:**
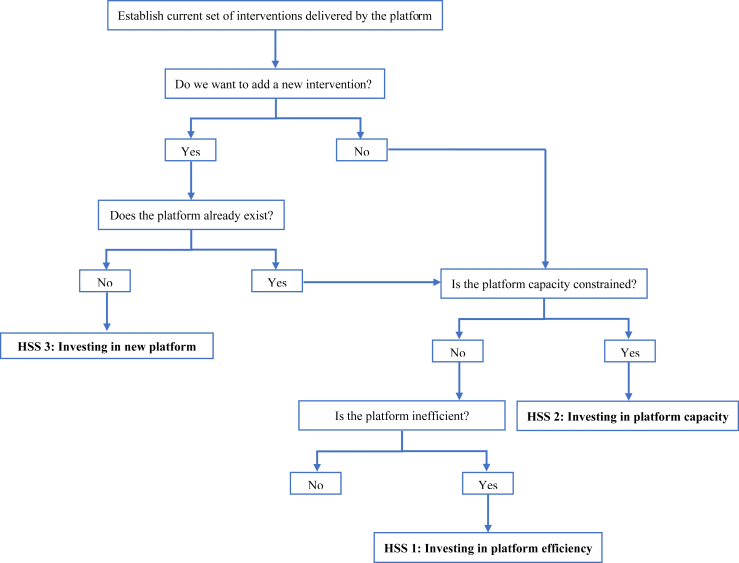
Decision steps for investments into health system strengthening.

We envisage that the next steps from our research may be the development of decision rules or a ‘checklist’ of the type or types of HSS that may accompany major adoption decisions. This would not only provide structure to the decision problem and break it down into distinct steps, but would also illustrate what kind of data and empirical evidence is required to help with informed decision making on HSS. Fig. 1 is a simple illustration of the kind of steps that decision makers need to go through when investing in HSS. A key feature of the decision problem is the comparison of economic value of investments in HSS versus a new intervention, or investments in HSS that are made alongside introduction of the new intervention. Our framework in this paper can be thought of as a first attempt to provide the conceptual framework behind such a checklist. In the longer term, we need more empirical analysis that sheds light on the role that platforms play in promoting the cost-effectiveness of different interventions, more methodological research on theoretically robust modelling frameworks for planning, and more experience with using such models in the field in real health service planning contexts. Only then can we hope to see a truly rigorous approach to the economic evaluation of HSS investments.

## Funding

International Decision Support Initiative (www.idsihealth.org), Bill & Melinda Gates Foundation, Department for International Development (UK). KH received additional funding from the National Institute for Health Research Health Protection Research Unit (NIHR HPRU) in Modelling Methodology at Imperial College London in partnership with Public Health England, and by the MRC Centre for Outbreak Analysis and Modelling (funding reference MR/K010174/1B). The views expressed are those of the authors and not necessarily those of the International Decision Support Initiative, the NHS, the NIHR or the Department of Health. RM received funding from the NIHR.

## Conflicts of interest

Alec Morton received personal fees from Office of Health Economics and AstraZeneca unrelated to this work. The remaining authors have no conflicts of interests to declare.
